# The impact of the COVID-19 pandemic on scientific research in the life sciences

**DOI:** 10.1371/journal.pone.0263001

**Published:** 2022-02-09

**Authors:** Massimo Riccaboni, Luca Verginer

**Affiliations:** 1 AXES, IMT School for Advanced Studies Lucca, Lucca, Italy; 2 Chair of Systems Design D-MTEC, ETH Zürich, Zurich, Switzerland; University of Rennes 1, FRANCE

## Abstract

The COVID-19 outbreak has posed an unprecedented challenge to humanity and science. On the one side, public and private incentives have been put in place to promptly allocate resources toward research areas strictly related to the COVID-19 emergency. However, research in many fields not directly related to the pandemic has been displaced. In this paper, we assess the impact of COVID-19 on world scientific production in the life sciences and find indications that the usage of medical subject headings (MeSH) has changed following the outbreak. We estimate through a difference-in-differences approach the impact of the start of the COVID-19 pandemic on scientific production using the PubMed database (3.6 Million research papers). We find that COVID-19-related MeSH terms have experienced a 6.5 fold increase in output on average, while publications on unrelated MeSH terms dropped by 10 to 12%. The publication weighted impact has an even more pronounced negative effect (-16% to -19%). Moreover, COVID-19 has displaced clinical trial publications (-24%) and diverted grants from research areas not closely related to COVID-19. Note that since COVID-19 publications may have been fast-tracked, the sudden surge in COVID-19 publications might be driven by editorial policy.

## Introduction

The COVID-19 pandemic has mobilized the world scientific community in 2020, especially in the life sciences [1, 2]. In the first three months after the pandemic, the number of scientific papers about COVID-19 was fivefold the number of articles on H1N1 swine influenza [3]. Similarly, the number of clinical trials related to COVID-19 prophylaxis and treatments skyrocketed [4]. Thanks to the rapid mobilization of the world scientific community, COVID-19 vaccines have been developed in record time. Despite this undeniable success, there is a rising concern about the negative consequences of COVID-19 on clinical trial research, with many projects being postponed [5–7]. According to Evaluate Pharma, clinical trials were one of the pandemic’s first casualties, with a record number of 160 studies suspended for reasons related to COVID-19 in April 2020 [8, 9] reporting a total of 1,200 trials suspended as of July 2020. As a consequence, clinical researchers have been impaired by reduced access to healthcare research infrastructures. Particularly, the COVID-19 outbreak took a tall on women and early-career scientists [10–13]. On a different ground, Shan and colleagues found that non-COVID-19-related articles decreased as COVID-19-related articles increased in top clinical research journals [14]. Fraser and coworker found that COVID-19 preprints received more attention and citations than non-COVID-19 preprints [1]. More recently, Hook and Porter have found some early evidence of ‘covidisation’ of academic research, with research grants and output diverted to COVID-19 research in 2020 [15]. How much should scientists switch their efforts toward SARS-CoV-2 prevention, treatment, or mitigation? There is a growing consensus that the current level of ‘covidisation’ of research can be wasteful [4, 5, 16].

Against this background, in this paper, we investigate if the COVID-19 pandemic has induced a shift in biomedical publications toward COVID-19-related scientific production. The objective of the study is to show that scientific articles listing *covid-related* Medical Subject Headings (MeSH) when compared against *covid-unrelated* MeSH have been partially displaced. Specifically, we look at several indicators of scientific production in the life sciences before and after the start of the COVID-19 pandemic: (1) number of papers published, (2) impact factor weighted number of papers, (3) opens access, (4) number of publications related to clinical trials, (5) number of papers listing grants, (6) number of papers listing grants existing before the pandemic. Through a natural experiment approach, we analyze the impact of the pandemic on scientific production in the life sciences. We consider COVID-19 an unexpected and unprecedented exogenous source of variation with heterogeneous effects across biomedical research fields (i.e., MeSH terms).

Based on the difference in difference results, we document the displacement effect that the pandemic has had on several aspects of scientific publishing. The overall picture that emerges from this analysis is that there has been a profound realignment of priorities and research efforts. This shift has displaced biomedical research in fields not related to COVID-19.

The rest of the paper is structured as follows. First, we describe the data and our measure of relatedness to COVID-19. Next, we illustrate the difference-in-differences specification we rely on to identify the impact of the pandemic on scientific output. In the results section, we present the results of the difference-in-differences and network analyses. We document the sudden shift in publications, grants and trials towards COVID-19-related MeSH terms. Finally, we discuss the findings and highlight several policy implications.

## Materials and methods

The present analysis is based primarily on PubMed and the Medical Subject Headings (MeSH) terminology. This data is used to estimate the effect of the start of the COVID 19 pandemic via a difference in difference approach. This section is structured as follows. We first introduce the data and then the econometric methodology. This analysis is not based on a pre-registered protocol.

### Data

#### Selection of biomedical publications

We rely on PubMed, a repository with more than 34 million biomedical citations, for the analysis. Specifically, we analyze the daily updated files up to 31/06/2021, extracting all publications of type ‘Journal Article’. For the principal analysis, we consider 3,638,584 papers published from January 2019 to December 2020. We also analyze 11,122,017 papers published from 2010 onwards to identify the earliest usage of a grant and infer if it was new in 2020. We use the SCImago journal ranking statistics to compute the impact factor weighted number (IFWN) of papers in a given field of research. To assign the publication date, we use the ‘electronically published’ dates and, if missing, the ‘print published’ dates.

#### Medical subject headings

We rely on the Medical Subject Headings (MeSH) terminology to approximate narrowly defined biomedical research fields. This terminology is a curated medical vocabulary, which is manually added to papers in the PubMed corpus. The fact that MeSH terms are manually annotated makes this terminology ideal for classification purposes. However, there is a delay between publication and annotation, on the order of several months. To address this delay and have the most recent classification, we search for all 28 425 MeSH terms using PubMed’s ESearch utility and classify paper by the results. The specific API endpoint is https://eutils.ncbi.nlm.nih.gov/entrez/eutils/esearch.fcgi, the relevant scripts are available with the code. For example, we assign the term ‘Ageusia’ (MeSH ID D000370) to all papers listed in the results of the ESearch API. We apply this method to the whole period (January 2019—December 2020) and obtain a mapping from papers to the MeSH terms. For every MeSH term, we keep track of the year they have been established. For instance, COVID-19 terms were established in 2020 (see Table 1): in January 2020, the WHO recommended 2019-nCoV and 2019-nCoV acute respiratory disease as provisional names for the virus and disease. The WHO issued the official terms COVID-19 and SARS-CoV-2 at the beginning of February 2020. By manually annotating publications, all publications referring to COVID-19 and SARS-CoV-2 since January 2020 have been labelled with the related MeSH terms. Other MeSH terms related to COVID-19, such as coronavirus, for instance, have been established years before the pandemic (see Table 2). We proxy MeSH term usage via search terms using the PubMed EUtilities API; this means that we are not using the hand-labelled MeSH terms but rather the PubMed search results. This means that the accuracy of the MeSH term we assign to a given paper is not perfect. In practice, this means that we have assigned more MeSH terms to a given term than a human annotator would have.

**Table 1:** MeSH terms used as the focal COVID-19 terms.

Unique ID	MeSH Heading	Established	Relatedness (*σ*)	Papers in 2020
D000086382	COVID-19	2020	1.0	72,058
D000086402	SARS-CoV-2	2020	1.0	46,076
D000086742	COVID-19 Testing	2020	1.0	6,095
D000086663	COVID-19 Vaccines	2020	1.0	2,578
D000087123	COVID-19 Nucleic Acid Testing	2020	1.0	1,744
D000087124	COVID-19 Serological Testing	2020	1.0	386

**Table 2:** Top 20 terms most often used listing also a COVID-19 MeSH term. The list contains only terms with at least 100 publications in 2020.

Unique ID	MeSH Heading	Established	Similarity (*σ*)	Papers 2020
D017934	Coronavirus	1994	0.999	55,256
D000073640	Betacoronavirus	2018	0.999	36,909
D018352	Coronavirus Infections	1994	0.999	46,754
D003333	Coronaviridae Infections	1977	0.999	45,536
D003332	Coronaviridae	1974	0.999	37,364
D004752	Coronavirus, Turkey	1991	0.999	854
D030341	Nidovirales Infections	2002	0.998	41,991
D028381	Nidovirales	2002	0.998	37,370
D045473	SARS Virus	2003	0.997	9403
D028962	Coronavirus OC43, Human	2002	0.995	114
D011024	Pneumonia, Viral	1966	0.991	45,741
D058873	Pandemics	2011	0.983	40,919
D000073638	Alphacoronavirus	2018	0.967	188
D017758	Inf. Dis. Transm., Patient-to-Professional	1994	0.964	916
D017757	Inf. Dis. Transm., Professional-to-Patient	1994	0.964	916
D045169	Severe Acute Respiratory Syndrome	2003	0.963	10,371
D000370	Ageusia	1991	0.958	176
D012141	Respiratory Tract Infections	1966	0.917	49,974
D004196	Disease Outbreaks	1968	0.915	43,745
D002268	Carboxypeptidases	1966	0.903	1,383

#### Clinical trials and publication types

We classify publications using PubMed’s ‘PublicationType’ field in the XML baseline files (There are 187 publication types, see https://www.nlm.nih.gov/mesh/pubtypes.html). We consider a publication to be related to a clinical trial if it lists any of the following descriptors:

- D016430: Clinical Trial
- D017426: Clinical Trial, Phase I
- D017427: Clinical Trial, Phase II
- D017428: Clinical Trial, Phase III
- D017429: Clinical Trial, Phase IV
- D018848: Controlled Clinical Trial
- D065007: Pragmatic Clinical Trial
- D000076362: Adaptive Clinical Trial
- D000077522: Clinical Trial, Veterinary

In our analysis of the impact of COVID-19 on publications related to clinical trials, we only consider MeSH terms that are associated at least once with a clinical trial publication over the two years. We apply this restriction to filter out MeSH terms that are very unlikely to be relevant for clinical trial types of research.

#### Open access

We proxy the availability of a journal article to the public, i.e., open access, if it is available from PubMed Central. PubMed Central archives full-text journal articles and provides free access to the public. Note that the copyright license may vary across participating publishers. However, the text of the paper is for all effects and purposes freely available without requiring subscriptions or special affiliation.

#### Grants

We infer if a publication has been funded by checking if it lists any grants. We classify grants as either ‘old’, i.e. existed before 2019, or ‘new’, i.e. first observed afterwards. To do so, we collect all grant IDs for 11,122,017 papers from 2010 on-wards and record their first appearance. This procedure is an indirect inference of the year the grant has been granted. The basic assumption is that if a grant number has not been listed in any publication since 2010, it is very likely a new grant. Specifically, an old grant is a grant listed since 2019 observed at least once from 2010 to 2018.

Note that this procedure is only approximate and has a few shortcomings. Mistyped grant numbers (e.g. ‘1234-M JPN’ and ‘1234-M-JPN’) could appear as new grants, even though they existed before, or new grants might be classified as old grants if they have a common ID (e.g. ‘Grant 1’). Unfortunately, there is no central repository of grant numbers and the associated metadata; however, there are plans to assign DOI numbers to grants to alleviate this problem (See https://gitlab.com/crossref/open_funder_registry for the project).

#### Impact factor weighted publication numbers (IFWN)

In our analysis, we consider two measures of scientific output. First, we simply count the number of publications by MeSH term. However, since journals vary considerably in terms of impact factor, we also weigh the number of publications by the impact factor of the venue (e.g., journal) where it was published. Specifically, we use the SCImago journal ranking statistics to weigh a paper by the impact factor of the journal it appears in. We use the ‘citation per document in the past two years’ for 45,230 ISSNs. Note that a journal may and often has more than one ISSN, i.e., one for the printed edition and one for the online edition. SCImago applies the same score for a venue across linked ISSNs.

For the impact factor weighted number (IFWN) of publication per MeSH terms, this means that all publications are replaced by the impact score of the journal they appear in and summed up.

#### COVID-19-relatedness

To measure how closely related to COVID-19 is a MeSH term, we introduce an index of relatedness to COVID-19. First, we identify the focal COVID-19 terms, which appeared in the literature in 2020 (see Table 1). Next, for all other pre-existing MeSH terms, we measure how closely related to COVID-19 they end up being.

Our aim is to show that MeSH terms that *existed before and are related* have experienced a sudden increase in the number of (impact factor weighted) papers.

We define a MeSH term’s COVID-19 relatedness as the conditional probability that, given its appearance on a paper, also one of the focal COVID-19 terms listed in Table 1 are present. In other words, the relatedness of a MeSH term is given by the probability that a COVID-19 MeSH term appears alongside. Since the focal COVID-19 terms did not exist before 2020, we estimate this measure only using papers published since January 2020. Formally, we define COVID-19-relatedness (*σ*) as in Eq (1), where C is the set of papers listing a COVID-19 MeSH term and $M_{i}$ is the set of papers listing MeSH term *i*.

$$\begin{array}{c} \sigma_{i}=P\left(C|M_{i}\right)=\frac{|M_{i}\cap C|}{|M_{i}|} \end{array}$$ (1)

Intuitively we can read this measure as: what is the probability in 2020 that a COVID-19 MeSH term is present given that we chose a paper with MeSH term *i*? For example, given that in 2020 we choose a paper dealing with “Ageusia” (i.e., Complete or severe loss of the subjective sense of taste), there is a 96% probability that this paper also lists COVID-19, see Table 1.

Note that a paper listing a related MeSH term does not imply that that paper is doing COVID-19 research, but it implies that one of the MeSH terms listed is often used in COVID-19 research.

#### Variables

In sum, in our analysis, we use the following variables:

1. Papers: Number of papers by MeSH term;
2. Impact: Impact factor weighted number of papers by MeSH term;
3. PMC: Papers listed in PubMed central by MeSH term, as a measure of Open Access publications;
4. Trials: number of publications of type “Clinical Trial” by MeSH term;
5. Grants: number of papers with at least one grant by MeSH term;
6. Old Grants: number of papers listing a grant that has been observed between 2010 and 2018, by MeSH term;

## Methods

### Difference-in-differences

The difference-in-differences (DiD) method is an econometric technique to imitate an experimental research design from observation data, sometimes referred to as a quasi-experimental setup. In a randomized controlled trial, subjects are randomly assigned either to the treated or the control group. Analogously, in this natural experiment, we assume that medical subject headings (MeSH) have been randomly assigned to be either treated (related) or not treated (unrelated) by the pandemic crisis.

Before the COVID, for a future health crisis, the set of potentially impacted medical knowledge was not predictable since it depended on the specifics of the emergency. For instance, ageusia (loss of taste), a medical concept existing since 1991, became known to be a specific symptom of COVID-19 only after the pandemic.

Specifically, we exploit the COVID-19 as an unpredictable and exogenous shock that has deeply affected the publication priorities for biomedical scientific production, as compared to the situation before the pandemic. In this setting, COVID-19 is the treatment, and the identification of this new human coronavirus is the event. We claim that treated MeSH terms, i.e., MeSH terms related to COVID-19, have experienced a sudden increase in terms of scientific production and attention. In contrast, research on untreated MeSH terms, i.e., MeSH terms not related to COVID-19, has been displaced by COVID-19. Our analysis compares the scientific output of COVID-19 related and unrelated MeSH terms before and after January 2020.

Consider the simple regression model in Eq (2). We have an outcome *Y* and dummy variable *P* identifying the period as *before* the event *P* = 0 and *P* = 1 as *after* the event. Additionally, we have a dummy variable identifying an observation belonging to the treated group (*G* = 1) or control (*G* = 0) group.

$$\begin{array}{c} Y=\beta_{0}+\beta_{1}P+\beta_{2}G+\beta_{3}P*G+\epsilon \end{array}$$ (2)

In our case, some of the terms turn out to be related to COVID-19 in 2020, whereas most of the MeSH terms are not closely related to COVID-19.

Thus *β*_1_ identifies the overall effect on the control group after the event, *β*_2_ the difference across treated and control groups before the event (i.e. the first difference in DiD) and finally the effect on the treated group after the event, net of the first difference, *β*_3_. This last parameter identifies the treatment effect on the treated group netting out the pre-treatment difference.

For the DiD to have a causal interpretation, it must be noted that pre-event, the trends of the two groups should be parallel, i.e., the common trend assumption (CTA) must be satisfied. We will show that the CTA holds in the results section.

To specify the DiD model, we need to define a period before and after the event and assign a treatment status or level of exposure to each term.

#### Before and after

The pre-treatment period is defined as January 2019 to December 2019. The post-treatment period is defined as the months from January 2020 to December 2020. We argue that the state of biomedical research was similar in those two years, apart from the effect of the pandemic.

#### Treatment status and exposure

The treatment is determined by the COVID-19 relatedness index *σ*_*i*_ introduced earlier. Specifically, this number indicates the likelihood that COVID-19 will be a listed MeSH term, given that we observe the focal MeSH term *i*. To show that the effect becomes even stronger the closer related the subject is, and for ease of interpretation, we also discretize the relatedness value into three levels of treatment. Namely, we group MeSH terms with a *σ* between, 0% to 20%, 20% to 80% and 80% to 100%. The choice of alternative grouping strategies does not significantly affect our results. Results for alternative thresholds of relatedness can be computed using the available source code. We complement the dichotomized analysis by using the treatment intensity (relatedness measure *σ*) to show that the result persists.

#### Panel regression

In this work, we estimate a random effects panel regression where the units of analysis are 28 318 biomedical research fields (i.e. MeSH terms) observed over time before and after the COVID-19 pandemic. The time resolution is at the monthly level, meaning that for each MeSH term, we have 24 observations from January 2019 to December 2020.

The basic panel regression with continuous treatment follows a similar setup as Eq (2) but with MeSH term random effects and monthly fixed effects.

$$\begin{array}{c} Y_{it}=\beta_{0}+\beta_{1}P_{t}+\gamma_{1}\sigma_{i}+\delta_{1}\sigma_{i}\times P_{t}+\sum_{k=2}^{24}\alpha_{t}I\left(t=k\right)+\nu_{i}+u_{it} \end{array}$$ (3)

The outcome variable *Y*_*it*_ identifies the outcome at time *t* (i.e., month), for MeSH term *i*. As before, *P*_*t*_ identifies the period with *P*_*t*_ = 0 if the month is before January 2020 and *P*_*t*_ = 1 if it is on or after this date. In (3), the treatment level is measure by the relatedness to COVID-19 (*σ*_*i*_), where again the *γ*_1_ identifies pre-trend (constant) differences and *δ*_1_ the overall effect.

As mentioned before, to highlight that the effect is not linear but increases with relatedness, we split *σ* into three groups: from 0% to 20%, 20% to 80% and 80% to 100%. In the three-level treatment specification, the number of treatment levels (*G*_*i*_) is 3; hence we have two *γ* parameters. Note that *I*(⋅) is the indicator function, which is 1 if the argument is true, and 0 otherwise.

$$\begin{array}{c} Y_{it}=\beta_{0}+\beta_{1}P_{t}+\sum_{k=1}^{2}\gamma_{k}I\left(G_{i}=k\right)+\sum_{k=1}^{2}\delta_{k}I\left(G_{i}=k\right)\times P_{t}+\sum_{k=2}^{24}\alpha_{t}I\left(t=k\right)+\nu_{i}+u_{it} \end{array}$$ (4)

In total, we estimate six coefficients. As before, the *δ*_*l*_ coefficient identifies the DiD effect.

#### Verifying the Common Trend Assumption (CTA)

To show that the pre-event trends are parallel and that the effect on publication activity is only visible from January 2020, we estimate a panel regression with each month modelled as a different event. Specifically, we estimate the following model.

$$\begin{array}{c} Y_{it}=\beta_{0}+\sum_{k=2}^{24}\alpha_{t}I\left(t=k\right)+\sum_{k=1}^{2}\gamma_{i}I\left(G_{i}=k\right)+\sum_{k=2}^{24}\sum_{j=1}^{2}\delta_{ij}I\left(t=k\right)I\left(G_{i}=j\right) \end{array}$$ (5)

We show that the CTA holds for this model by comparing the pre-event trends of the control group to the treated groups (COVID-19 related MeSH terms). Namely, we show that the pre-event trends of the control group are the same as the pre-event trends of the treated group.

### Co-occurrence analysis

To investigate if the pandemic has caused a reconfiguration of research priorities, we look at the MeSH term co-occurrence network. Precisely, we extract the co-occurrence network of all 28,318 MeSH terms as they appear in the 3.3 million papers. We considered the co-occurrence networks of 2018, 2019 and 2020. Each node represents a MeSH term in these networks, and a link between them indicates that they have been observed at least once together. The weight of the edge between the MeSH terms is given by the number of times those terms have been jointly observed in the same publications.

Medical language is hugely complicated, and this simple representation does not capture the intricacies, subtle nuances and, in fact, meaning of the terms. Therefore, we do not claim that we can identify *how* the actual usage of MeSH terms has changed from this object, but rather that it has. Nevertheless, the co-occurrence graph captures rudimentary relations between concepts. We argue that absent a shock to the system, their basic usage patterns, change in importance (within the network) would essentially be the same from year to year. However, if we find that the importance of terms changes more than expected in 2020, it stands to reason that there have been some significant changes.

To show that that MeSH usage has been affected, we compute for each term in the years 2018, 2019 and 2020 their PageRank centrality [17]. The PageRank centrality tells us how likely a random walker traversing a network would be found at a given node if she follows the weights of the empirical edges (i.e., co-usage probability). Specifically, for the case of the MeSH co-occurrence network, this number represents how often an annotator at the National Library of Medicine would assign that MeSH term following the observed general usage patterns. It is a simplistic measure to capture the complexities of biomedical research. Nevertheless, it captures far-reaching interdependence across MeSH terms as the measure uses the whole network to determine the centrality of every MeSH term. A sudden change in the rankings and thus the position of MeSH terms in this network suggests that a given research subject has risen as it is used more often with other important MeSH terms (or vice versa).

To show that COVID-19-related research has profoundly impacted the way MeSH terms are used, we compute for each MeSH term the change in its PageRank centrality (*p*_*it*_).

$$\begin{array}{c} g_{i}\left(t\right)=\frac{p_{i}\left(t\right)-p_{i}\left(t-1\right)}{p_{i}\left(t-1\right)} \end{array}$$ (6)

We then compare the growth for each MeSH *i* term in *g*_*i*_(2019), i.e. before the the COVID-19 pandemic, with the growth after the event (*g*_*i*_(2020)).

### Publication growth

To estimate growth in scientific output, we compute the year over year growth in the number of the impact weighted number of publications per MeSH. Specifically, we measure the year by year growth as defined below, where *m* is the impact weighted number of publications at time *t*.

$$\begin{array}{c} g_{i}\left(t\right)=\frac{m_{i}\left(t\right)-m_{i}\left(t-1\right)}{m_{i}\left(t-1\right)} \end{array}$$ (7)

## Results

### Changes in output and COVID-19 relatedness

Before we show the regression results, we provide descriptive evidence that publications from 2019 to 2020 have drastically increased. By showing that this growth correlates strongly with a MeSH term’s COVID-19 relatedness (*σ*), we demonstrate that (1) *σ* captures an essential aspect of the growth dynamics and (2) highlight the meteoric rise of highly related terms.

We look at the year over year growth in the number of the impact weighted number of publications per MeSH term from 2018 to 2019 and 2019 to 2020 as defined in the methods section.

**Fig 1:**
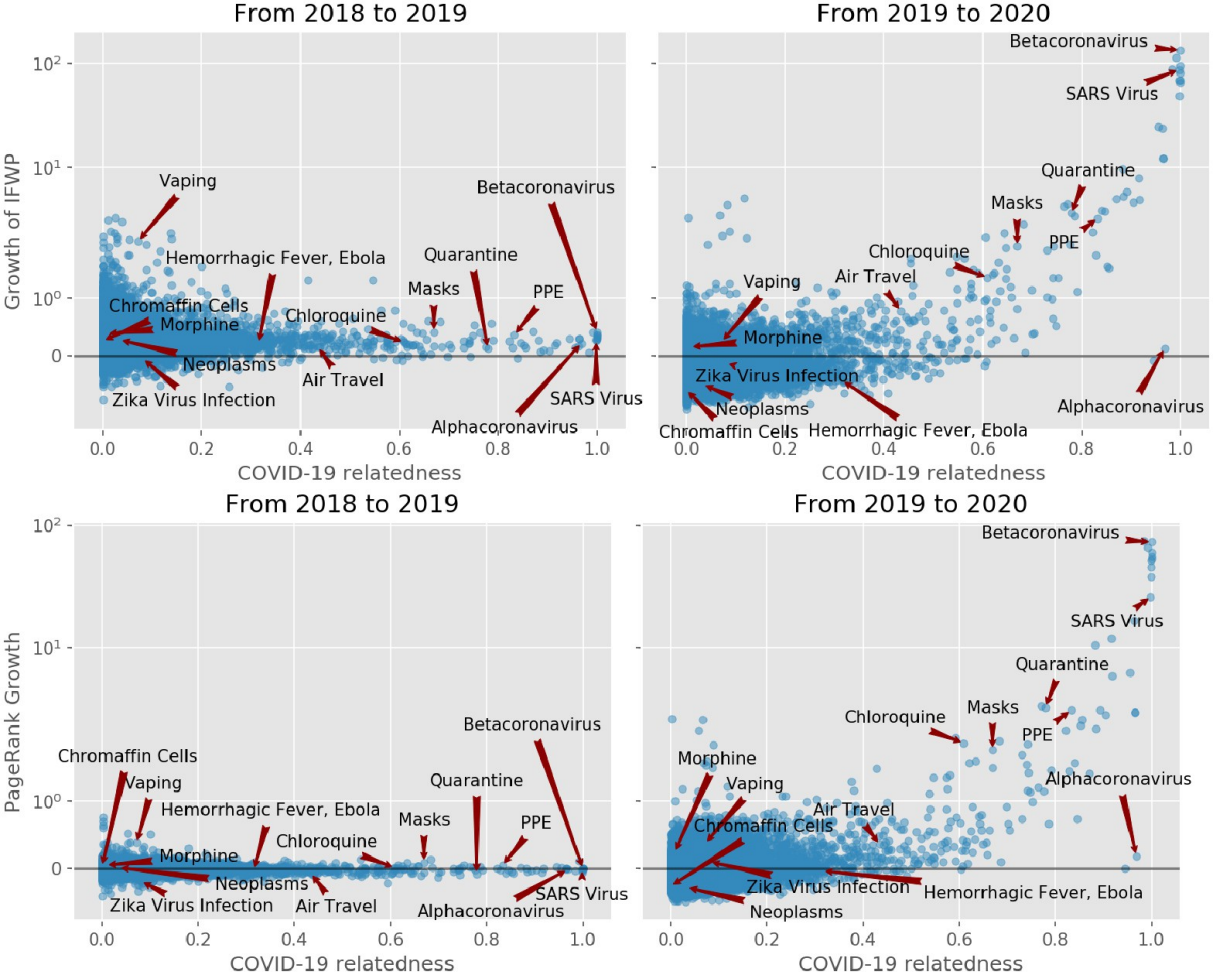
Above: Impact factor weighted publication number (IFWN) growth per MeSH term from 2018 to 2019 and from 2019 to 2020. Each dot represents, a MeSH term. The y axis (growth) is in symmetric log scale. The x axis shows the COVID-19 relatedness, *σ*. Note that the position of the dots on the x-axis is the same in the two plots. Below: MeSH term importance gain (PageRank) and their COVID-19 relatedness.

Fig 1 shows the yearly growth of the impact weighted number of publications per MeSH term. By comparing the growth of the number of publications from the years 2018, 2019 and 2020, we find that the impact factor weighted number of publications has increased by up to a factor of 100 compared to the previous year for Betacoronavirus, one of the most closely related to COVID-19 MeSH term.

Fig 1, first row, reveals how strongly correlated the growth in the IFWN of publication is to the term’s COVID-19 relatedness. For instance, we see that the term ‘Betacoronavirus’ skyrocketed from 2019 to 2020, which is expected given that SARS-CoV-2 is a species of the genus. Conversely, the term ‘Alphacoronavirus’ has not experienced any growth given that it is twin a genus of the Coronaviridae family, but SARS-CoV-2 is not one of its species. Note also the fast growth in the number of publications dealing with ‘Quarantine’. Moreover, MeSH terms that grew significantly from 2018 to 2019 and were not closely related to COVID-19, like ‘Vaping’, slowed down in 2020. From the graph, the picture emerges that publication growth is correlated with COVID-19 relatedness *σ* and that the growth for less related terms slowed down.

To show that the usage pattern of MeSH terms has changed following the pandemic, we compute the PageRank centrality using graph-tool [18] as discussed in the Methods section.

Fig 1, second row, shows the change in the PageRank centrality of the MeSH terms after the pandemic (2019 to 2020, right plot) and before (2018 to 2019, left plot). If there were no change in the general usage pattern, we would expect the variance in PageRank changes to be narrow across the two periods, see (left plot). However, PageRank scores changed significantly more from 2019 to 2020 than from 2018 to 2019, suggesting that there has been a reconfiguration of the network.

To further support this argument, we carry out a DiD regression analysis.

### Common trends assumption

**Fig 2:**
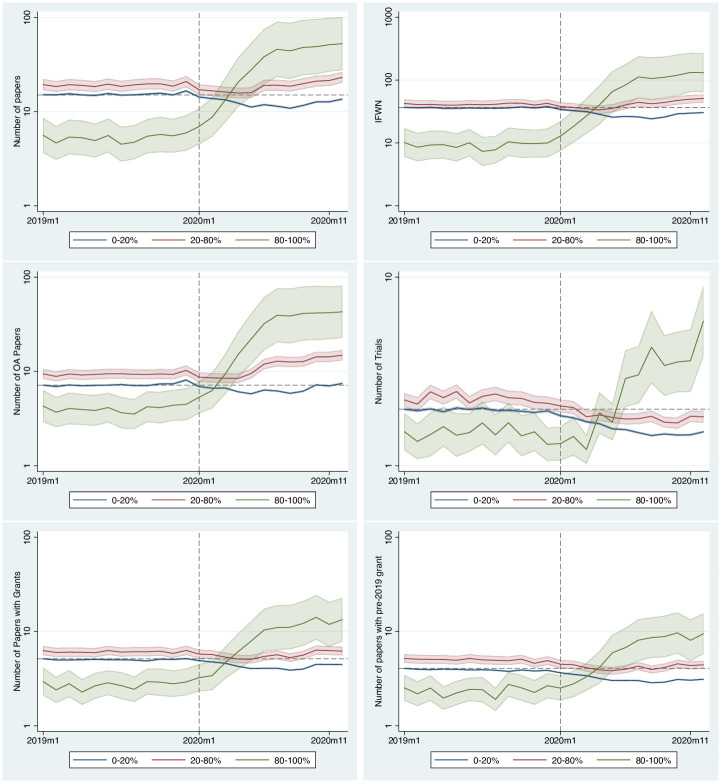
Predicted number of papers, impact factor weighted number of papers, open access papers, papers related to clinical trials, total number of papers with grants and older grants (before 2019) per month. The y axis is in log scale. The dashed vertical line identifies January 2020. The dashed horizontal line shows the publications in January 2019 for the 0–20% group before the event. This line highlights that the drop happens after the event. The bands around the lines indicate the 95% confidence interval of the predicted values. The results are the output of the Stata margins command.

As discussed in the Methods section, we need to show that the CTA assumption holds for the DiD to be defined appropriately. We do this by estimating for each month the number of publications and comparing it across treatment groups. This exercise also serves the purpose of a placebo test. By assuming that each month could have potentially been the event’s timing (i.e., the outbreak), we show that January 2020 is the most likely timing of the event. The regression table, as noted earlier, contains over 70 estimated coefficients, hence for ease of reading, we will only show the predicted outcome per month by group (see Fig 2). The full regression table with all coefficients is available in the S1 Table.

Fig 2 shows the predicted number per outcome variable obtained from the panel regression model. These predictions correspond to the predicted value per relatedness group using the regression parameters estimated via the linear panel regression. The bands around the curves are the 95% confidence intervals.

All outcome measures depict a similar trend per month. Before the event (i.e., January 2020), there is a common trend across all groups. In contrast, after the event, we observe a sudden rise for the outcomes of the COVID-19 related treated groups (green and red lines) and a decline in the outcomes for the unrelated group (blue line). Therefore, we can conclude that the CTA assumption holds.

### Regression results

Table 3 shows the DiD regression results (see Eq (3)) for the selected outcome measures: number of publications (Papers), impact factor weighted number of publications (Impact), open access (OA) publications, clinical trial related publications, and publications with existing grants.

**Table 3:** Random effects difference in difference regression with continuous treatment variable.

	(1)	(2)	(3)	(4)	(5)	(6)
	ln(Papers)	ln(Impact)	ln(PMC)	ln(Trials)	ln(Grants)	ln(Old Grants)
After COVID-19	-0.129***	-0.214***	0.016***	-0.272***	-0.153***	-0.271***
	(-27.56)	(-34.23)	(3.56)	(-56.64)	(-36.05)	(-65.47)
Relatedness (*σ*)	2.852***	2.813***	2.787***	1.308***	2.330***	2.374***
	(13.88)	(12.33)	(15.50)	(11.61)	(14.60)	(15.50)
After COVID-19 × Relatedness (*σ*)	0.961***	1.237***	1.203***	0.058	0.494***	0.332***
	(12.49)	(14.83)	(17.23)	(1.11)	(8.10)	(5.73)
Constant	2.606***	3.485***	1.863***	0.630***	1.547***	1.312***
	(197.00)	(224.53)	(159.76)	(74.34)	(142.71)	(129.07)
Month Effects	Yes	Yes	Yes	Yes	Yes	Yes
Observations	679632	679632	679632	417552	679632	679632
MeSH Terms	28,318	28,318	28,318	17,398	28,318	28,318
R2 within	0.090	0.056	0.051	0.102	0.050	0.087
R2 between	0.023	0.018	0.030	0.018	0.020	0.023
R2 overall	0.026	0.021	0.031	0.032	0.022	0.028

t statistics in parentheses, Std. Err. adjusted by MeSH-id. All outcome variables are in natural log.

* *p* < 0.05,

** *p* < 0.01,

*** *p* < 0.001

Table 3 shows results for the discrete treatment level version of the DiD model (see Eq (4)).

Note that the outcome variable is in natural log scale; hence to get the effect of the independent variable, we need to exponentiate the coefficient. For values close to 0, the effect is well approximated by the percentage change of that magnitude.

In both specifications we see that the least related group, drops in the number of publications between 10% and 13%, respectively (first row of Tables 3 and 4, exp(−0.102) ≈ 0.87). In line with our expectations, the increase in the number of papers published by MeSH term is positively affected by the relatedness to COVID-19. In the discrete model (row 2), we note that the number of documents with MeSH terms with a COVID-19 relatedness between 20 and 80% grows by 18% and highly related terms by a factor of approximately 6.6 (exp(1.88)). The same general pattern can be observed for the impact weighted publication number, i.e., Model (2). Note, however, that the drop in the impact factor weighted output is more significant, reaching -19% for COVID-19 unrelated publications, and related publications growing by a factor of 8.7. This difference suggests that there might be a bias to publish papers on COVID-19 related subjects in high impact factor journals.

**Table 4:** Random effects difference in difference regression with discrete treatment levels.

	(1)	(2)	(3)	(4)	(5)	(6)
	ln(Papers)	ln(Impact)	ln(PMC)	ln(Trials)	ln(Grants)	ln(Old Grants)
After COVID-19	-0.102***	-0.178***	0.049***	-0.271***	-0.139***	-0.263***
	(-24.82)	(-31.26)	(12.60)	(-60.84)	(-36.22)	(-69.46)
20%≤*σ* ≤ 80%	0.228***	0.128	0.260***	0.144***	0.192***	0.243***
	(3.38)	(1.67)	(4.33)	(3.52)	(3.47)	(4.63)
80%≤*σ* ≤ 100%	-1.069***	-1.373***	-0.587**	-0.278*	-0.620***	-0.511***
	(-5.09)	(-5.37)	(-3.13)	(-2.40)	(-3.92)	(-3.47)
After COVID-19 ×(20% ≤ *σ* ≤ 80%)	0.170***	0.236***	0.279***	0.005	0.073***	0.048***
	(12.91)	(15.78)	(21.92)	(0.45)	(6.69)	(4.47)
After COVID-19 ×(80% ≤ *σ* ≤ 100%)	1.880***	2.163***	1.822***	0.753***	1.254***	1.140***
	(10.05)	(10.54)	(10.14)	(7.14)	(8.58)	(8.58)
Constant	2.716***	3.599***	1.968***	0.689***	1.636***	1.401***
	(226.29)	(256.68)	(182.06)	(86.00)	(160.31)	(145.47)
Month Effects	Yes	Yes	Yes	Yes	Yes	Yes
Observations	679632	679632	679632	417552	679632	679632
MeSH Terms	28,318	28,318	28,318	17,398	28,318	28,318
R2 within	0.096	0.058	0.052	0.105	0.054	0.091
R2 between	0.001	0.000	0.002	0.001	0.001	0.001
R2 overall	0.005	0.005	0.005	0.018	0.004	0.008

t statistics in parentheses, Std. Err. adjusted by MeSH-id. All outcome variables are in natural log. *σ* is the MeSH term relatedness to COVID-19.

* *p* < 0.05,

** *p* < 0.01,

*** *p* < 0.001

By looking at the number of open access publications (PMC), we note that the least related group has not been affected negatively by the pandemic. However, the number of COVID-19 related publications has drastically increased for the most COVID-19 related group by a factor of 6.2. Note that the substantial increase in the number of papers available through open access is in large part due to journal and editorial policies to make preferentially COVID research immediately available to the public.

Regarding the number of clinical trial publications, we note that the least related group has been affected negatively, with the number of publications on clinical trials dropping by a staggering 24%. At the same time, publications on clinical trials for COVID-19-related MeSH have increased by a factor of 2.1. Note, however, that the effect on clinical trials is not significant in the continuous regression. The discrepancy across Tables 3 and 4 highlights that, especially for trials, the effect is not linear, where only the publications on clinical trials closely related to COVID-19 experiencing a boost.

It has been reported [19] that while the number of clinical trials registered to treat or prevent COVID-19 has surged with 179 new registrations in the second week of April 2020 alone. Only a few of these have led to publishable results in the 12 months since [20]. On the other hand, we find that clinical trial publications, considering related MeSH (but not COVID-19 directly), have had significant growth from the beginning of the pandemic. These results are not contradictory. Indeed counting the number of clinical trial publications listing the exact COVID-19 MeSH term (D000086382), we find 212 publications. While this might seem like a small number, consider that in 2020 only 8,485 publications were classified as clinical trials; thus, targeted trials still made up 2.5% of *all clinical trials in 2020*. So while one might doubt the effectiveness of these research efforts, it is still the case that by sheer number, they represent a significant proportion of all publications on clinical trials in 2020. Moreover, COVID-19 specific Clinical trial publications in 2020, being a delayed signal of the actual trials, are a lower bound estimate on the true number of such clinical trials being conducted. This is because COVID-19 studies could only have commenced in 2020, whereas other studies had a head start. Thus our reported estimates are conservative, meaning that the true effect on actual clinical trials is likely larger, not smaller.

Research funding, as proxied by the number of publications with grants, follows a similar pattern, but notably, COVID-19-related MeSH terms list the same proportion of grants established before 2019 as other unrelated MeSH terms, suggesting that grants which were not designated for COVID-19 research have been used to support COVID-19 related research. Overall, the number of publications listing a grant has dropped. Note that this should be because the number of publications overall in the unrelated group has dropped. However, we note that the drop in publications is 10% while the decline in publications with at least one grant is 15%. This difference suggests that publications listing grants, which should have more funding, are disproportionately COVID-19 related papers. To further investigate this aspect, we look at whether the grant was old (pre-2019) or appeared for the first time in or after 2019. It stands to reason that an old grant (pre-2019) would not have been granted for a project dealing with the pandemic. Hence we would expect that COVID-19 related MeSH terms to have a lower proportion of old grants than the unrelated group. In models (6) in Table 4 we show that the number of old grants for the unrelated group drops by 13%. At the same time, the number of papers listing old grants (i.e., pre-2019) among the most related group increased by a factor of 3.1. Overall, these results suggest that COVID-19 related research has been funded largely by pre-existing grants, even though a specific mandate tied to the grants for this use is unlikely.

## Discussion

The scientific community has swiftly reallocated research efforts to cope with the COVID-19 pandemic, mobilizing knowledge across disciplines to find innovative solutions in record time. We document this both in terms of changing trends in the biomedical scientific output and the usage of MeSH terms by the scientific community. The flip side of this sudden and energetic prioritization of effort to fight COVID-19 has been a sudden contraction of scientific production in other relevant research areas. All in all, we find strong support to the hypotheses that the COVID-19 crisis has induced a sudden increase of research output in COVID-19 related areas of biomedical research. Conversely, research in areas not related to COVID-19 has experienced a significant drop in overall publishing rates and funding.

Our paper contributes to the literature on the impact of COVID-19 on scientific research: we corroborate previous findings about the surge of COVID-19 related publications [1–3], partially displacing research in COVID-19 unrelated fields of research [4, 14], particularly research related to clinical trials [5–7]. The drop in trial research might have severe consequences for patients affected by life-threatening diseases since it will delay access to new and better treatments. We also confirm the impact of COVID-19 on open access publication output [1]; also, this is milder than traditional outlets. On top of this, we provide more robust evidence on the impact weighted effect of COVID-19 and grant financed research, highlighting the strong displacement effect of COVID-19 on the allocation of financial resources [15]. We document a substantial change in the usage patterns of MeSH terms, suggesting that there has been a reconfiguration in the way research terms are being combined. MeSH terms highly related to COVID-19 were peripheral in the MeSH usage networks before the pandemic but have become central since 2020. We conclude that the usage patterns have changed, with COVID-19 related MeSH terms occupying a much more prominent role in 2020 than they did in the previous years.

We also contribute to the literature by estimating the effect of COVID-19 on biomedical research in a natural experiment framework, isolating the specific effects of the COVID-19 pandemic on the biomedical scientific landscape. This is crucial to identify areas of public intervention to sustain areas of biomedical research which have been neglected during the COVID-19 crisis. Moreover, the exploratory analysis on the changes in usage patterns of MeSH terms, points to an increase in the importance of covid-related topics in the broader biomedical research landscape.

Our results provide compelling evidence that research related to COVID-19 has indeed displaced scientific production in other biomedical fields of research not related to COVID-19, with a significant drop in (impact weighted) scientific output related to non-COVID-19 and a marked reduction of financial support for publications not related to COVID-19 [4, 5, 16]. The displacement effect is persistent to the end of 2020. As vaccination progresses, we highlight the urgent need for science policy to re-balance support for research activity that was put on pause because of the COVID-19 pandemic.

We find that COVID-19 dramatically impacted clinical research. Reactivation of clinical trials activities that have been postponed or suspended for reasons related to COVID-19 is a priority that should be considered in the national vaccination plans. Moreover, since grants have been diverted and financial incentives have been targeted to sustain COVID-19 research leading to an excessive entry in COVID-19-related clinical trials and the ‘covidisation’ of research, there is a need to reorient incentives to basic research and otherwise neglected or temporally abandoned areas of biomedical research. Without dedicated support in the recovery plans for neglected research of the COVID-19 era, there is a risk that more medical needs will be unmet in the future, possibly exacerbating the shortage of scientific research for orphan and neglected diseases, which do not belong to COVID-19-related research areas.

### Limitations

Our empirical approach has some limits. First, we proxy MeSH term usage via search terms using the PubMed EUtilities API. This means that the accuracy of the MeSH term we assign to a given paper is not fully validated. More time is needed for the completion of manually annotated MeSH terms. Second, the timing of publication is not the moment the research has been carried out. There is a lead time between inception, analysis, write-up, review, revision, and final publication. This delay varies across disciplines. Nevertheless, given that the surge in publications happens around the alleged event date, January 2020, we are confident that the publication date is a reasonable yet imperfect estimate of the timing of the research. Third, several journals have publicly declared to fast-track COVID-19 research. This discrepancy in the speed of publication of COVID-19 related research and other research could affect our results. Specifically, a surge or displacement could be overestimated due to a lag in the publication of COVID-19 unrelated research. We alleviate this bias by estimating the effect considering a considerable time after the event (January 2020 to December 2020). Forth, on the one hand, clinical Trials may lead to multiple publications. Therefore we might overestimate the impact of COVID-19 on the number of clinical trials. On the other hand, COVID-19 publications on clinical trials lag behind, so the number of papers related COVID-19 trials is likely underestimated. Therefore, we note that the focus of this paper is scientific publications on clinical trials rather than on actual clinical trials. Fifth, regarding grants, unfortunately, there is no unique centralized repository mapping grant numbers to years, so we have to proxy old grants with grants that appeared in publications from 2010 to 2018. Besides, grant numbers are free-form entries, meaning that PubMed has no validation step to disambiguate or verify that the grant number has been entered correctly. This has the effect of classifying a grant as new even though it has appeared under a different name. We mitigate this problem by using a long period to collect grant numbers and catch many spellings of the same grant, thereby reducing the likelihood of miss-identifying a grant as new when it existed before. Still, unless unique identifiers are widely used, there is no way to verify this.

So far, there is no conclusive evidence on whether entry into COVID-19 has been excessive. However, there is a growing consensus that COVID-19 has displaced, at least temporally, scientific research in COVID-19 unrelated biomedical research areas. Even though it is certainly expected that more attention will be devoted to the emergency during a pandemic, the displacement of biomedical research in other fields is concerning. Future research is needed to investigate the long-run structural consequences of the COVID-19 crisis on biomedical research.

## Supporting information

S1 Table Common trend assumption (CTA) regression table. Full regression table with all controls and interactions.
(PDF)
