# Supplementary material for: The impact of the COVID-19 pandemic on scientific research in the life sciences
Source: PLoS One. 2022 Feb 9;17(2):e0263001. doi: 10.1371/journal.pone.0263001 (PMC8827464; doi:10.1371/journal.pone.0263001)
Supplement: S1 Table — Full regression table with all controls and interactions. (PDF) [file pone.0263001.s001.pdf]

Supplementary material for the paper: The impact of the COVID-19 pandemic on scientific research in the life sciences

January 21, 2022

Table S 1: **Common trend assumption (CTA) Regression Table.** Full Regression table with all controls and interactions.

	(1)<br>ln(Papers)	(2)<br>ln(Impact)	(3)<br>ln(PMC)	(4)<br>ln(Trials)	(5)<br>ln(Grants)	(6)<br>ln(Old Grants)
2019-03	-0.001	-0.007	-0.032***	-0.019***	-0.029***	-0.022***
	(-0.41)	(-1.51)	(-10.32)	(-4.72)	(-9.40)	(-7.12)
2019-03	0.024***	0.011*	0.005	0.004	-0.030***	-0.033***
	(8.29)	(2.40)	(1.76)	(1.09)	(-9.58)	(-10.69)
2019-04	-0.004	-0.023***	-0.014***	-0.031***	-0.023***	-0.018***
	(-1.30)	(-4.89)	(-4.60)	(-7.87)	(-7.48)	(-5.85)
2019-05	-0.018***	-0.022***	-0.005	0.017***	-0.016***	-0.025***
	(-6.34)	(-4.78)	(-1.56)	(4.31)	(-5.19)	(-8.24)
2019-06	0.031***	-0.014**	0.001	-0.003	-0.023***	-0.037***
	(10.54)	(-3.04)	(0.37)	(-0.74)	(-7.39)	(-11.92)
2019-07	-0.009**	-0.023***	0.015***	0.015***	-0.023***	-0.035***
	(-3.12)	(-4.81)	(4.86)	(3.85)	(-7.49)	(-11.26)
2019-08	-0.002	-0.028***	-0.010**	-0.016***	-0.033***	-0.046***
	(-0.70)	(-5.86)	(-3.05)	(-4.06)	(-10.52)	(-14.97)
2019-09	0.017***	-0.015**	-0.010**	-0.014***	-0.053***	-0.073***
	(5.90)	(-3.12)	(-3.13)	(-3.65)	(-17.02)	(-23.64)
2019-10	0.038***	0.022***	0.032***	-0.025***	-0.009**	-0.040***
	(13.19)	(4.83)	(10.35)	(-6.19)	(-3.02)	(-12.88)
2019-11	-0.003	-0.009*	0.032***	-0.039***	-0.017***	-0.064***
	(-1.10)	(-1.98)	(10.29)	(-9.79)	(-5.33)	(-20.80)
2019-12	0.094***	0.037***	0.126***	-0.020***	0.004	-0.049***
	(32.00)	(8.06)	(39.21)	(-5.03)	(1.35)	(-15.79)
2020-01	-0.056***	-0.062***	-0.034***	-0.080***	-0.046***	-0.108***
	(-18.39)	(-12.75)	(-10.47)	(-20.01)	(-14.28)	(-33.69)
2020-02	-0.093***	-0.113***	-0.072***	-0.105***	-0.077***	-0.147***
	(-29.85)	(-23.12)	(-21.85)	(-26.06)	(-23.69)	(-45.46)
2020-03	-0.121***	-0.146***	-0.069***	-0.153***	-0.110***	-0.177***
	(-37.45)	(-29.29)	(-20.33)	(-36.88)	(-32.68)	(-53.00)
2020-04	-0.206***	-0.249***	-0.161***	-0.177***	-0.187***	-0.247***
	(-59.90)	(-47.87)	(-44.64)	(-41.43)	(-52.39)	(-70.53)
2020-05	-0.303***	-0.352***	-0.210***	-0.240***	-0.240***	-0.297***
	(-84.07)	(-65.94)	(-55.94)	(-54.85)	(-63.99)	(-80.55)

2020-06	-0.243***	-0.323***	-0.117***	-0.248***	-0.232***	-0.294***
	(-62.33)	(-58.38)	(-30.06)	(-55.86)	(-60.38)	(-79.10)
2020-07	-0.280***	-0.343***	-0.141***	-0.286***	-0.231***	-0.299***
	(-72.11)	(-61.31)	(-35.81)	(-62.75)	(-59.39)	(-78.76)
2020-08	-0.330***	-0.413***	-0.199***	-0.321***	-0.279***	-0.351***
	(-80.45)	(-72.08)	(-48.01)	(-67.97)	(-68.10)	(-88.07)
2020-09	-0.261***	-0.348***	-0.147***	-0.303***	-0.252***	-0.338***
	(-62.18)	(-59.68)	(-35.28)	(-65.03)	(-60.64)	(-83.50)
2020-10	-0.175***	-0.229***	0.008*	-0.315***	-0.137***	-0.271***
	(-44.50)	(-40.85)	(2.23)	(-68.44)	(-36.63)	(-72.35)
2020-11	-0.172***	-0.204***	-0.018***	-0.312***	-0.141***	-0.290***
	(-42.62)	(-35.23)	(-4.88)	(-67.63)	(-36.89)	(-76.78)
2020-12	-0.107***	-0.185***	0.043***	-0.276***	-0.142***	-0.266***
	(-25.85)	(-31.95)	(10.93)	(-61.49)	(-36.54)	(-69.51)
$20\% \leq \sigma \leq 80\%$	0.249***	0.151	0.276***	0.113**	0.198***	0.241***
	(3.67)	(1.93)	(4.53)	(2.66)	(3.49)	(4.45)
$80\% \leq \sigma \leq 100\%$	-0.991***	-1.282***	-0.510**	-0.274*	-0.557**	-0.476**
	(-4.55)	(-4.79)	(-2.58)	(-2.38)	(-3.27)	(-2.99)
2019-02 $\times$ $20\% \leq \sigma \leq 80\%$	-0.047***	-0.032	-0.029*	-0.031	-0.015	0.005
	(-3.34)	(-1.40)	(-2.01)	(-1.54)	(-1.03)	(0.35)
2019-02 $\times$ $80\% \leq \sigma \leq 100\%$	-0.186***	-0.161*	-0.116*	-0.101	-0.175***	-0.123**
	(-4.57)	(-2.19)	(-2.55)	(-1.80)	(-3.47)	(-2.84)
2019-03 $\times$ $20\% \leq \sigma \leq 80\%$	-0.026	-0.046*	-0.008	0.095***	-0.007	0.013
	(-1.87)	(-2.05)	(-0.56)	(4.47)	(-0.48)	(0.92)
2019-03 $\times$ $80\% \leq \sigma \leq 100\%$	-0.068	-0.100	-0.063	-0.039	-0.021	0.028
	(-1.58)	(-1.21)	(-1.46)	(-0.58)	(-0.52)	(0.61)
2019-04 $\times$ $20\% \leq \sigma \leq 80\%$	-0.017	-0.035	-0.013	0.059**	-0.016	-0.005
	(-1.21)	(-1.48)	(-0.92)	(2.90)	(-1.10)	(-0.36)
2019-04 $\times$ $80\% \leq \sigma \leq 100\%$	-0.057	-0.054	-0.070	0.092*	-0.234***	-0.233***
	(-1.17)	(-0.69)	(-1.57)	(1.99)	(-4.19)	(-3.82)
2019-05 $\times$ $20\% \leq \sigma \leq 80\%$	-0.030*	-0.040	-0.008	0.086***	-0.033*	-0.018
	(-2.24)	(-1.77)	(-0.53)	(4.03)	(-2.28)	(-1.19)
2019-05 $\times$ $80\% \leq \sigma \leq 100\%$	-0.112*	-0.156	-0.101	-0.060	-0.087	-0.101*
	(-2.19)	(-1.85)	(-1.81)	(-0.84)	(-1.80)	(-2.53)
2019-06 $\times$ $20\% \leq \sigma \leq 80\%$	-0.020	-0.020	0.001	-0.033	0.027	0.037*
	(-1.37)	(-0.84)	(0.07)	(-1.47)	(1.72)	(2.32)
2019-06 $\times$ $80\% \leq \sigma \leq 100\%$	-0.035	0.007	-0.036	-0.009	-0.006	-0.001
	(-0.69)	(0.09)	(-0.61)	(-0.19)	(-0.10)	(-0.01)
2019-07 $\times$ $20\% \leq \sigma \leq 80\%$	-0.035*	-0.026	-0.012	0.029	-0.013	0.001
	(-2.52)	(-1.17)	(-0.78)	(1.43)	(-0.85)	(0.05)
2019-07 $\times$ $80\% \leq \sigma \leq 100\%$	-0.208***	-0.301***	-0.176***	0.092	-0.075	-0.010
	(-4.11)	(-3.84)	(-3.50)	(1.62)	(-1.51)	(-0.21)
2019-08 $\times$ $20\% \leq \sigma \leq 80\%$	-0.005	-0.003	-0.000	0.089***	0.005	0.002
	(-0.34)	(-0.13)	(-0.01)	(4.10)	(0.35)	(0.10)
2019-08 $\times$ $80\% \leq \sigma \leq 100\%$	-0.168**	-0.235*	-0.185***	-0.023	-0.156**	-0.236***
	(-3.13)	(-2.57)	(-3.34)	(-0.36)	(-2.64)	(-3.48)
2019-09 $\times$ $20\% \leq \sigma \leq 80\%$	-0.000	0.026	-0.004	0.041*	0.027	0.019
	(-0.01)	(1.17)	(-0.29)	(2.02)	(1.71)	(1.17)
2019-09 $\times$ $80\% \leq \sigma \leq 100\%$	-0.041	0.043	-0.007	0.124	0.051	0.157*

	(-0.68)	(0.45)	(-0.13)	(1.91)	(0.87)	(2.55)
2019-10 × 20% $\leq \sigma \leq$ 80%	-0.019	-0.013	-0.034*	0.044*	-0.003	0.024
	(-1.34)	(-0.55)	(-2.26)	(2.15)	(-0.17)	(1.58)
2019-10 × 80% $\leq \sigma \leq$ 100%	-0.018	-0.052	-0.069	-0.026	-0.005	0.040
	(-0.39)	(-0.80)	(-1.59)	(-0.42)	(-0.15)	(0.91)
2019-11 × 20% $\leq \sigma \leq$ 80%	-0.035*	-0.049*	-0.045**	0.009	-0.051***	-0.054***
	(-2.56)	(-2.15)	(-3.06)	(0.43)	(-3.40)	(-3.52)
2019-11 × 80% $\leq \sigma \leq$ 100%	-0.010	-0.028	-0.011	0.036	-0.036	-0.048
	(-0.14)	(-0.26)	(-0.14)	(0.70)	(-0.63)	(-0.87)
2019-12 × 20% $\leq \sigma \leq$ 80%	-0.020	-0.034	-0.040*	-0.020	0.002	-0.002
	(-1.38)	(-1.51)	(-2.49)	(-1.02)	(0.10)	(-0.10)
2019-12 × 80% $\leq \sigma \leq$ 100%	-0.037	-0.054	-0.083	-0.135*	-0.013	0.101
	(-0.54)	(-0.56)	(-1.27)	(-2.20)	(-0.23)	(1.69)
2020-01 × 20% $\leq \sigma \leq$ 80%	-0.070***	-0.050*	-0.047**	0.007	-0.038*	-0.031
	(-4.81)	(-2.03)	(-3.13)	(0.31)	(-2.35)	(-1.93)
2020-01 × 80% $\leq \sigma \leq$ 100%	0.272**	0.301*	0.250**	-0.066	0.142*	0.104
	(2.75)	(2.20)	(2.69)	(-1.06)	(1.99)	(1.54)
2020-02 × 20% $\leq \sigma \leq$ 80%	-0.060***	-0.030	-0.025	0.013	-0.018	-0.005
	(-3.97)	(-1.26)	(-1.60)	(0.57)	(-1.13)	(-0.29)
2020-02 × 80% $\leq \sigma \leq$ 100%	0.535***	0.704***	0.452***	0.047	0.223*	0.243**
	(4.21)	(4.43)	(3.71)	(0.75)	(2.46)	(3.08)
2020-03 × 20% $\leq \sigma \leq$ 80%	-0.062***	-0.049*	-0.036*	-0.052*	-0.047**	-0.048**
	(-3.77)	(-1.98)	(-2.16)	(-2.41)	(-2.73)	(-2.76)
2020-03 × 80% $\leq \sigma \leq$ 100%	0.979***	1.120***	0.849***	-0.065	0.569***	0.447***
	(6.25)	(5.80)	(6.06)	(-1.24)	(5.43)	(5.00)
2020-04 × 20% $\leq \sigma \leq$ 80%	-0.001	0.013	0.050**	-0.005	-0.019	-0.032
	(-0.06)	(0.50)	(2.77)	(-0.23)	(-1.08)	(-1.71)
2020-04 × 80% $\leq \sigma \leq$ 100%	1.505***	1.627***	1.423***	0.406***	0.917***	0.745***
	(7.87)	(7.38)	(8.05)	(5.33)	(6.75)	(6.07)
2020-05 × 20% $\leq \sigma \leq$ 80%	0.105***	0.176***	0.220***	0.027	0.028	-0.003
	(5.47)	(6.47)	(11.04)	(1.18)	(1.50)	(-0.17)
2020-05 × 80% $\leq \sigma \leq$ 100%	1.903***	2.223***	1.849***	0.357***	1.229***	1.154***
	(9.04)	(9.34)	(9.19)	(5.53)	(8.10)	(8.21)
2020-06 × 20% $\leq \sigma \leq$ 80%	0.224***	0.267***	0.356***	0.016	0.091***	0.033
	(10.68)	(9.24)	(16.32)	(0.67)	(4.71)	(1.75)
2020-06 × 80% $\leq \sigma \leq$ 100%	2.167***	2.433***	2.130***	0.897***	1.492***	1.277***
	(9.79)	(9.93)	(9.87)	(6.84)	(8.68)	(7.87)
2020-07 × 20% $\leq \sigma \leq$ 80%	0.264***	0.385***	0.451***	0.058*	0.130***	0.114***
	(12.63)	(13.25)	(20.64)	(2.42)	(6.73)	(5.94)
2020-07 × 80% $\leq \sigma \leq$ 100%	2.383***	2.747***	2.359***	0.979***	1.551***	1.465***
	(10.63)	(11.10)	(10.61)	(7.59)	(8.28)	(8.28)
2020-08 × 20% $\leq \sigma \leq$ 80%	0.292***	0.402***	0.488***	0.122***	0.108***	0.083***
	(13.42)	(13.48)	(21.71)	(5.32)	(5.36)	(4.25)
2020-08 × 80% $\leq \sigma \leq$ 100%	2.394***	2.760***	2.399***	1.352***	1.606***	1.577***
	(10.37)	(10.82)	(10.51)	(7.65)	(8.85)	(9.31)
2020-09 × 20% $\leq \sigma \leq$ 80%	0.284***	0.385***	0.447***	0.031	0.142***	0.118***
	(12.64)	(12.69)	(19.20)	(1.28)	(7.03)	(5.71)
2020-09 × 80% $\leq \sigma \leq$ 100%	2.411***	2.739***	2.409***	1.111***	1.672***	1.590***
	(10.69)	(11.13)	(10.79)	(7.56)	(9.10)	(9.06)

2020-10 × 20% ≤ σ ≤ 80%	0.259***	0.341***	0.408***	0.035	0.150***	0.142***
	(11.47)	(11.26)	(18.31)	(1.41)	(7.59)	(7.26)
2020-10 × 80% ≤ σ ≤ 100%	2.342***	2.695***	2.265***	1.165***	1.705***	1.614***
	(10.29)	(10.94)	(10.01)	(7.91)	(9.11)	(9.23)
2020-11 × 20% ≤ σ ≤ 80%	0.272***	0.360***	0.434***	0.113***	0.142***	0.119***
	(10.78)	(10.42)	(18.20)	(4.77)	(6.91)	(5.79)
2020-11 × 80% ≤ σ ≤ 100%	2.387***	2.774***	2.298***	1.180***	1.537***	1.451***
	(10.52)	(11.32)	(10.27)	(8.01)	(8.12)	(8.16)
2020-12 × 20% ≤ σ ≤ 80%	0.284***	0.366***	0.411***	0.071**	0.130***	0.113***
	(11.42)	(10.70)	(17.14)	(2.92)	(6.09)	(5.31)
2020-12 × 80% ≤ σ ≤ 100%	2.346***	2.745***	2.259***	1.626***	1.655***	1.588***
	(10.28)	(11.01)	(10.09)	(9.27)	(8.82)	(8.98)
Constant	2.715***	3.598***	1.968***	0.690***	1.636***	1.401***
	(226.07)	(256.29)	(181.82)	(85.95)	(160.07)	(145.25)
Observations	679,632	679,632	679,632	417,552	679,632	679,632
MeSH Terms	28,318	28,318	28,318	17,398	28,318	28,318
R2 within	0.103	0.063	0.061	0.108	0.057	0.095
R2 between	0.001	0.000	0.002	0.001	0.001	0.001
R2 overall	0.005	0.005	0.005	0.019	0.004	0.008

t statistics in parentheses, Std. Err. adjusted by MeSH-id. All outcome variables are in natural log.

* $p < 0.05$, ** $p < 0.01$, *** $p < 0.001$
